# Effect of wavelength and beam width on penetration in light-tissue interaction using computational methods

**DOI:** 10.1007/s10103-017-2317-4

**Published:** 2017-09-12

**Authors:** Caerwyn Ash, Michael Dubec, Kelvin Donne, Tim Bashford

**Affiliations:** 1 School of Applied Computing, University of Wales Trinity Saint David, Swansea, SA1 6ED UK; 2 The Christie NHS Foundation Trust, 550 Wilmslow Rd, Manchester, M20 4BX UK

**Keywords:** IPL, Laser, Monte Carlo, Penetration

## Abstract

Penetration depth of ultraviolet, visible light and infrared radiation in biological tissue has not previously been adequately measured. Risk assessment of typical intense pulsed light and laser intensities, spectral characteristics and the subsequent chemical, physiological and psychological effects of such outputs on vital organs as consequence of inappropriate output use are examined. This technical note focuses on wavelength, illumination geometry and skin tone and their effect on the energy density (fluence) distribution within tissue. Monte Carlo modelling is one of the most widely used stochastic methods for the modelling of light transport in turbid biological media such as human skin. Using custom Monte Carlo simulation software of a multi-layered skin model, fluence distributions are produced for various non-ionising radiation combinations. Fluence distributions were analysed using Matlab mathematical software. Penetration depth increases with increasing wavelength with a maximum penetration depth of 5378 μm calculated. The calculations show that a 10-mm beam width produces a fluence level at target depths of 1–3 mm equal to 73–88% (depending on depth) of the fluence level at the same depths produced by an infinitely wide beam of equal incident fluence. Meaning little additional penetration is achieved with larger spot sizes. Fluence distribution within tissue and thus the treatment efficacy depends upon the illumination geometry and wavelength. To optimise therapeutic techniques, light-tissue interactions must be thoroughly understood and can be greatly supported by the use of mathematical modelling techniques.

## Introduction

Human skin is the interface between man and his environment. One of the environmental factors it has to deal with is ultraviolet (UV) radiation. Ultraviolet radiation may cause negative effects such as erythema, skin ageing and skin cancer. The upper layer of skin, the epidermis, acts as a natural UV filter and may thicken or develop a stronger pigmentation as an adaption to excess UV exposure. On the other hand, UV radiation induces photo production of previtamin D_3_ in the skin and thus provides the major natural source for vitamin D_3_ for humans. Thus, it seems reasonable that skin has not evolved as a mere sun block but as a sun screen.

**Fig. 1:**
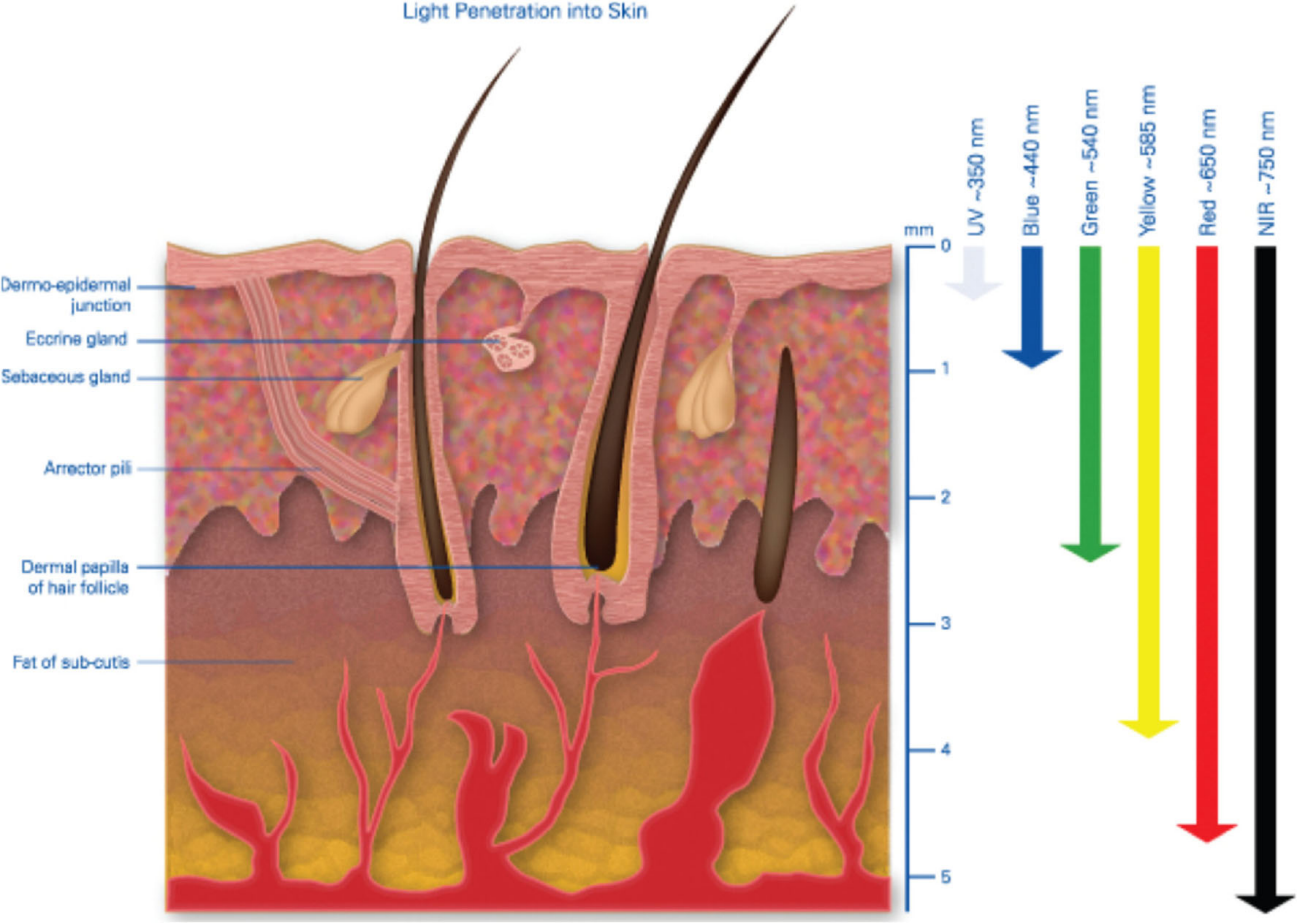
Light penetration into skin illustrating the depth to which wavelengths penetrate human skin. Red light is extinguished some 4–5 mm beneath the surface of the skin whereas ultraviolet hardly penetrates at all and blue barely 1 mm into tissue [8]

Red light penetrates deeper than blue light [1]. The reason is that skin consists of a range of chromophores which have scattering and absorption coefficients which are highly wavelength dependent [1–6]. The scattering properties of tissue are due to attenuation properties intrinsic to the chromophore and also to the size of the particles within the tissue which also governs the type of scattering that occurs, namely Mie or Rayleigh scattering [7]. Scattering leads to light dispersion in the tissue and the eventual reduction in the energy density with increasing depth [8]. Zhao and Fairchild looked at the transmission of laser light through tissues over a range of skin types and for laser wavelengths in the range 532–1064 nm and showed that 1064-nm light penetrated deepest into tissue [9] (Fig. 1).

Intense pulsed light (IPL) systems emit light in the wavelength range 400–1200 nm [10]. For wavelengths greater than 1000 nm, there is not a great amount of information regarding the penetration depth, and there is also little information regarding the photobiological effects at wavelengths greater than 1000 nm.

The wavelength of a therapeutic source therefore has a double importance, namely to ensure absorption of the incident photons in the target chromophores and to be able to do so at the depths at which these chromophores exist. The waveband in which the wavelength of the incident photons is located determines not only which part of the cell is the target but also the primary photo action. Wavelength is thus probably the single most important consideration in phototherapy, because without absorption, there can be no reaction.

For normally incident radiation, this regular reflectance is on the order of 4–7% [11]. The remaining 93–96% of the radiation entering the skin is either scattered or absorbed. The type of scattering is an elastic interaction between a photon and matter in which only the direction of propagation of the photon is altered. Elastic scattering results from inhomogeneity’s in the skin’s index of refraction corresponding to the physical boundaries of anatomical features, such as collagen fibres, and is characterised by the wavelength-dependent scattering coefficient, *μ*_s_ (*λ*) (cm^−1^). Once absorbed, the radiation is predominantly converted non-radiatively (i.e. without luminescence) into heat by the absorbing chromophore molecules. When there are pigmented tissue structures present that are more strongly absorbing at the laser wavelength than the surrounding tissue, e.g. hair shafts, the photons propagating randomly within the tissue will tend to be absorbed selectively by the pigmented structures. Thus, given enough deposited energy, selective photothermolysis of the pigmented structures will occur. Absorption by the pigmented structures or the surrounding tissue is characterised by their respective wavelength-dependent absorption coefficients, *μ*_a_ (*λ*) (cm^−1^).

A question that often appears throughout the literature asks: To what depth does optical light penetrate in skin tissue? This question is of importance when targeting treatment areas, optimising treatments, reducing risks and ensuring that the optical radiation does not reach vital organs and the foetus during pregnancy which may lead to complications. This computational investigation shows the effect of incident wavelength and beam width on penetration of human skin.

## Materials and method

**Fig. 2:**
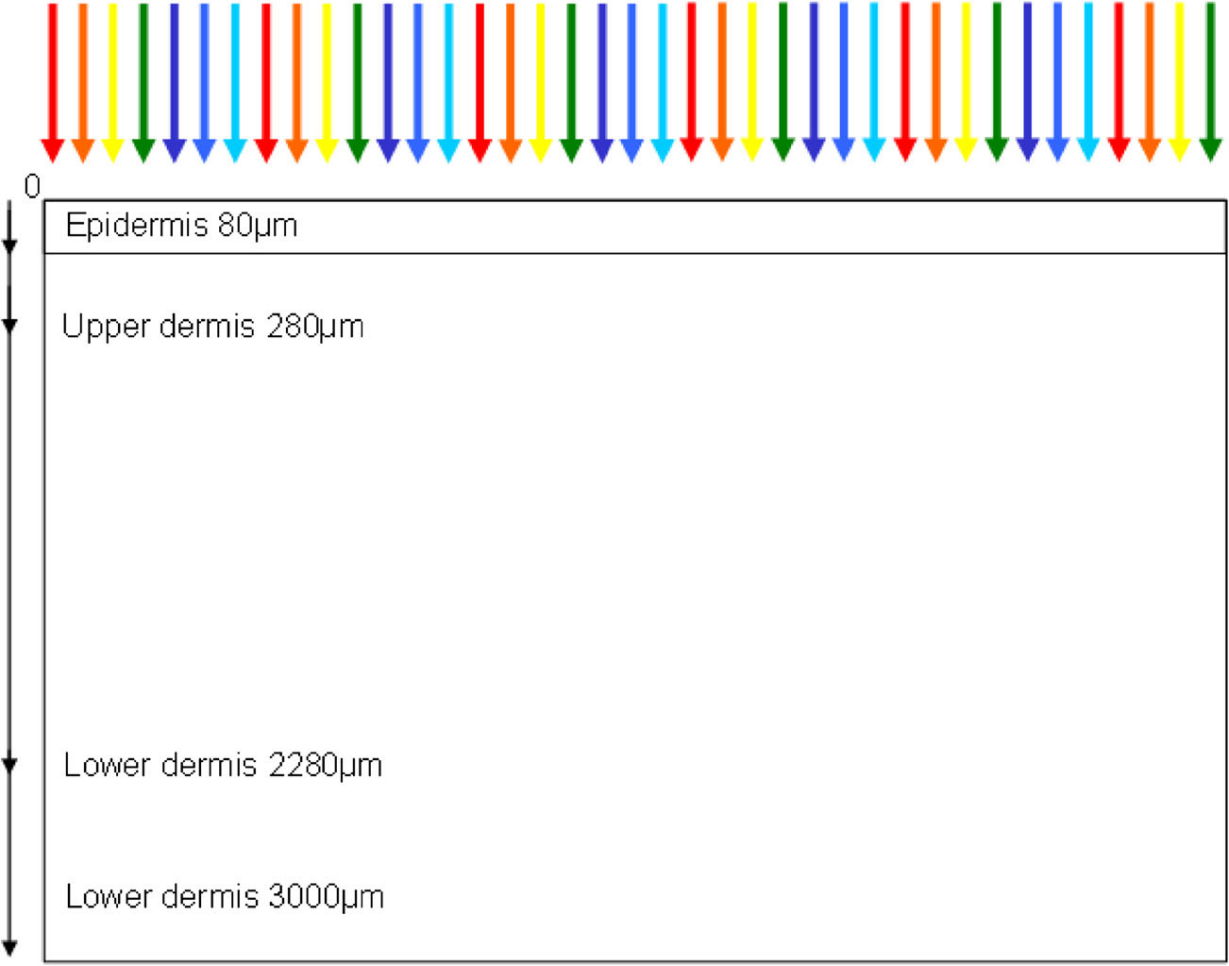
Diagram depicting the Monte Carlo model Cartesian geometry used

The 2-D mathematical simulation described here was previously utilised for a study modelling blood vessels [12], but was modified to simulate the 2-D penetration of photos into a homogeneous tissue matrix [13]. The authors of this study modelled the tissue matrix using a 5-μm cell size as a Cartesian coordinate, an 80-μm melanin rich epidermis with 5% melanin concentration and a melanin free dermis, as depicted in Fig. 2. The simulation is comprised of two interacting subroutines, a Monte Carlo photon distribution and a heat diffusion calculation to typical pulse structures as produced by an IPL system as shown in Fig. 2. The model was calibrated against thermography measurements of human skin exposed to IPL exposure. These measurements used various skin tones (Caucasian, Indian and Afro Caribbean) with ranging IPL exposure (2–20 J/cm^2^) at a known distance from skin. For this investigation, only the Monte Carlo section of photon distribution is used.

The calculation of the energy distribution was achieved by using a Monte Carlo process. This computer simulation allows observation of comparative data that cannot be performed in skin. The Monte Carlo simulation of photons is a numerical calculation repeated random sampling to obtain numerical results by probability distribution to compute the radiative transport in scattering and absorbing media.

**Fig. 3:**
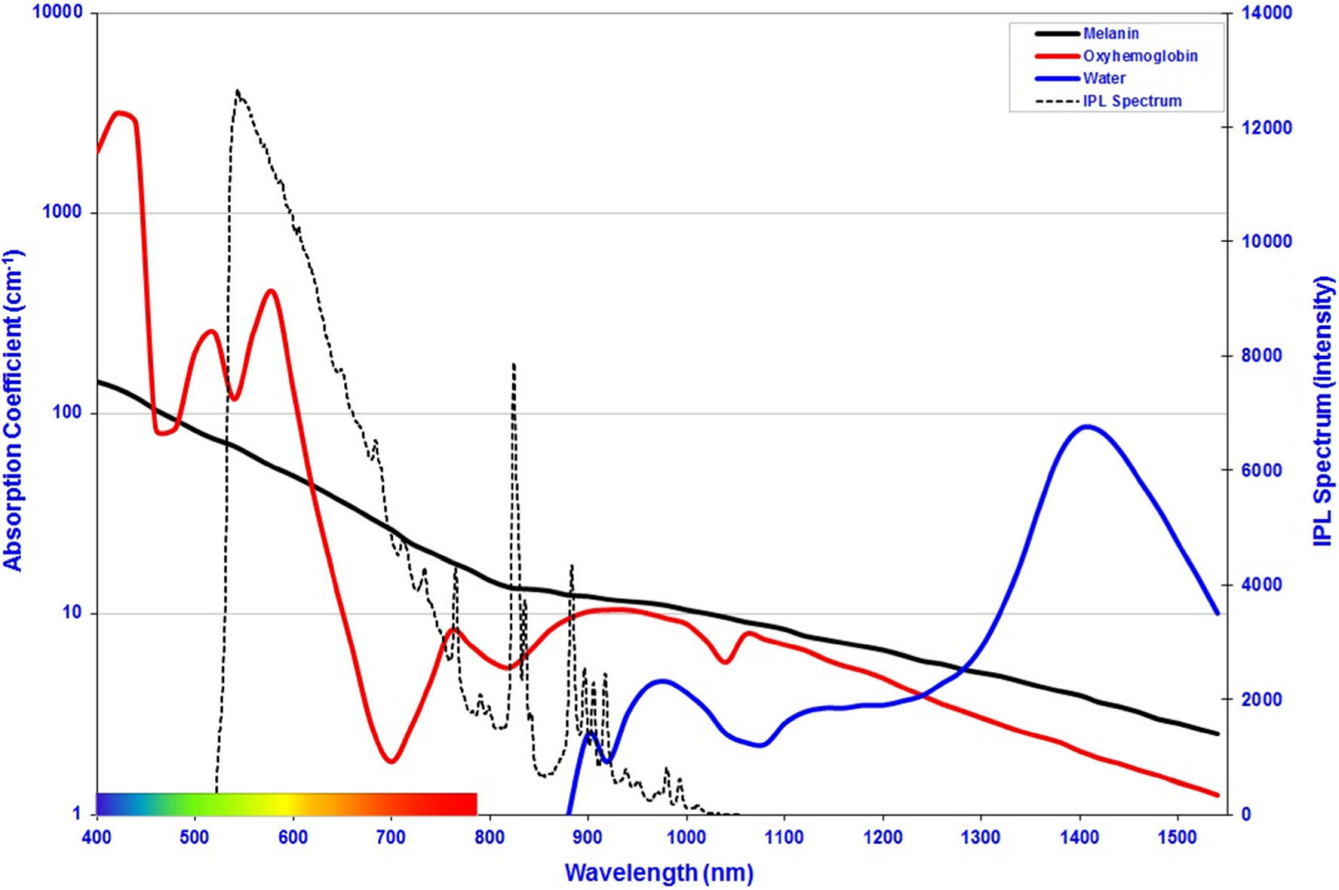
Absorption coefficients of melanin, oxyhaemoglobin and water. The IPL emission spectrum used for this evaluation is overlaid for reference [3]

In the computations of photon pathways, the absorption coefficients (*μ*_a_) provide energy conversion of the photon to the cell, the scattering coefficient (*μ*_s_) provides the angle of deflection between tissue cell interaction and the whole anisotropy factor (*g*) of each material at the specific wavelength is considered whereby light is forward scattered in tissue. These respective values were taken from the literature [14–19], and the respective absorption coefficients at these wavelengths are tabulated in Table 1 and shown in Fig. 3.

**Table 1:** Physical constants used for the various tissue layers used in this model [14–19]

	*k* (W m^−1^ K^−1^)	*ρ* (kg m^−3^)	*c* (J kg^−1^ K^−1^)	Refractive index	Anisotropy factor
Epidermis	0.5	1200	3600	1.34	0.789
Dermis	0.53	1200	3800	1.37	0.789
Hair	0.24	1210	3500	1.7	0.789

The wavelength dependency of the optical parameters of the IPL spectrum complicates mathematical modelling of light-tissue interaction for IPL, as compared to monochromatic lasers [20]. For comparison of system design and efficiency, the total radiant exposure of the 1 × 10^9^ photons that constitute the spectrum shown in Fig. 3 equals 10 J/cm^2^. When filtered broadband IPL is delivered to the subject’s skin, a fraction of the optical energy is reflected from the skin’s surface while the rest is scattered beneath the skin’s surface and is then absorbed in biological cells. Such absorption causes the hair shaft and hair bulb to heat up resulting in damage to the follicle, shedding of the hair and prevention of future regrowth.

A custom Monte Carlo simulation package was created by the School of Applied Computing, University of Wales Trinity Saint David, UK, and implemented on a personal computer. Within this Monte Carlo simulation, the absorption coefficient of melanin (*μ*_a_) is computed. The scattering coefficient (*μ*_s_) is measured from experimental techniques originating from Mie scattering due to collagen fibres and from Rayleigh scattering due to small tissue structures, respectively [21]. Scattering in tissue by photons is characterised by the Henyey-Greenstein scattering phase function [17, 22, 23] which is mathematically expressed in the form…

$$ P\left(\theta \right)=\frac{1-{g}^2}{\Big(1+{g}^2-2\ g\ \cos {\left(\theta \Big)\right)}^{3/2}} $$

**Fig. 4:**
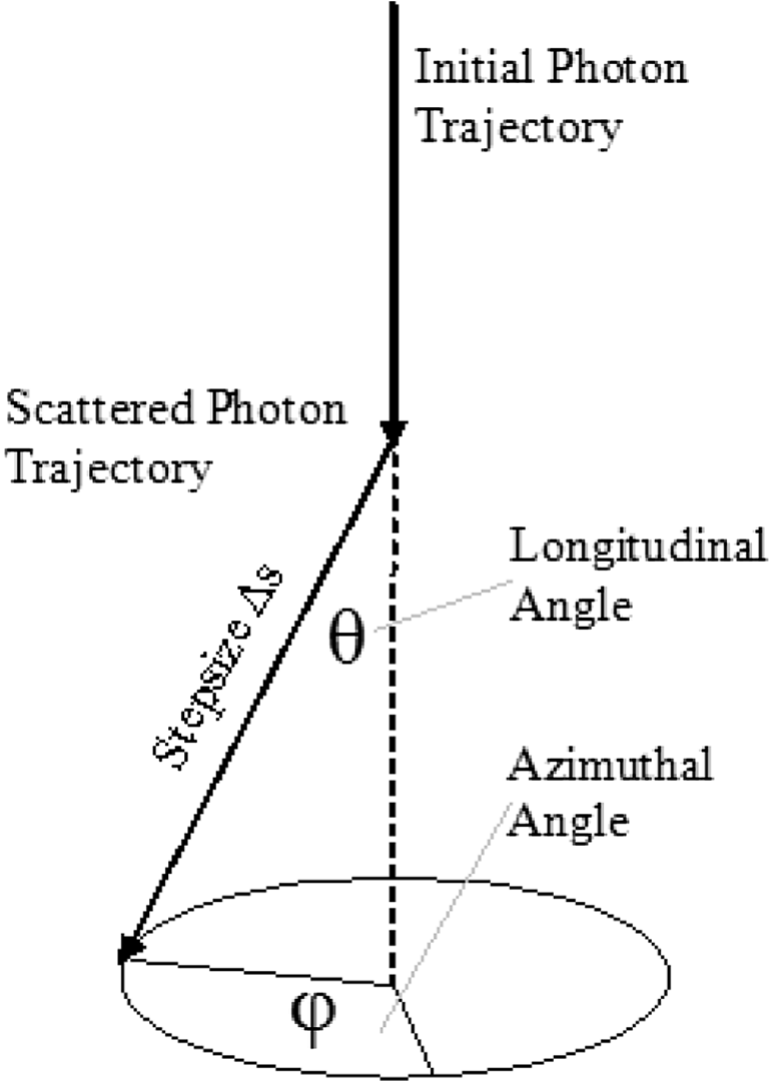
Deflection of a photon at a scattering point

The longitudinal angle of scattering, (*θ*), is characterised by the Henyey-Greenstein phase function, and since this phase function is forwardly biased, *θ* will be randomly influenced to reflect this characteristic. Applying the probability density function for the random number *R* to the above equation and solving for cos(*θ*) lead the following expression (Fig. 4).

$$ \mathrm{Cos}\left(\theta \right)=\frac{1}{2g}\left[1+{g}^2-{\left(\frac{1-{g}^2}{1-g+2 gR}\right)}^2\right]\ \mathrm{for}\ g\ne 0 $$

The azimuthal angle, *φ*, is uniformly distributed within the interval [0, 2π]. Its probability density function is constant and equals $\frac{1}{2\pi }$. Hence, *φ* takes the form:

$$ \varphi =2\ \pi\ R $$

A predefined number of photons are directed onto a skin model. These photons are given a weight (W), which is equal to 1. A photon starts at a boundary position with the tissue interface with a step size, anisotropy angle of rotation and a deflection angle. At each interaction, the photon delivers a portion of its energy, pre-set to values determined of tissue optical properties. The loss in energy at each of the on-going interaction sites is determined by the weight that the photon deposits. The change in weight is defined by the following equation [24–26].

$$ \Delta W=W\frac{\mu_{\mathrm{a}}}{\mu_{\mathrm{a}}+{\mu}_{\mathrm{s}}} $$

The photons are terminated when the value of Δ*W* is below a threshold. In this simulation, the photons are terminated when one hundredth of the original weight remained after multiple interactions. Then after this photon is deposited in its final position, a new photon is released and the process is repeated. A sufficient number of photons are required to generate an absorption energy density matrix for the defined tissue configuration for absorption of the IPL power output [24, 25, 27]. This energy density matrix is used with a factor of total energy output within this defined area [24, 25]. The absorbed energy at each Cartesian cell can be used as a heat source for thermal diffusion approximation. The time-dependant heat flow equation is reduced to its 2-D form and subsequently evaluated over the discretised domain using the Alternating Direction Implicit (ADI) method.

$$ \frac{\partial^2T}{\partial {y}^2}+\frac{\partial^2T}{\partial {z}^2}+\frac{H}{k}=\frac{1}{\alpha}\frac{\partial T}{\partial t} $$

Where *H* is the volumetric distribution of energy calculated in the Monte Carlo simulation and stored in number array, *y* and *z* are the radial and axial coordinates, respectively.

### Effect of wavelength

In order to obtain a relationship between the penetration depth and wavelength, Monte Carlo simulations were carried out for wavelengths in the range 300–750 nm in 50 nm increments. The default beam width of 30 mm was also used. Photon distributions were then extracted from the Monte Carlo model and input into a custom Matlab programme for data manipulation and more effective data analysis.

### Effect of beam width

In order to obtain a relationship between the penetration depth of light and the beam width of the treatment device used, Monte Carlo simulations were carried out for beam widths in the range of 1 to 40 mm. The simulations used 1 × 10^9^ photons with an epidermal melanin concentration of 4.3% (skin type 2) [28] on the top layer and a total dermis depth of 5560 μm.

For each of the beam widths used, a default IPL spectra was used (Fig. 3). Photon distributions were then extracted from the Monte Carlo model and input into a custom Matlab programme which provides a visual representation of the penetration depth of the light on a contour plot and also calculates the depth of penetration for and 1% values from the data matrices produced which then allowed beam width penetration depth relationships to be graphically represented.

Simulations were carried out for a range of beam widths in the range 1 to 40 mm, shown in Fig. 7. The results show that as the beam width is increased there is an increase in the depth of penetration, which can clearly be seen by the use of intensity contours, each representing a 1% fraction of the maximum intensity. The 1% fraction contours were chosen after experimentation with other values for penetration such as 1/*e* and 1/*e* ^2^ for a clear and clean graphical representation of the photo-distribution matrix. The figures also show the beam divergence as the light passes through the skin model.

## Results—effect of wavelength

**Fig. 5:**
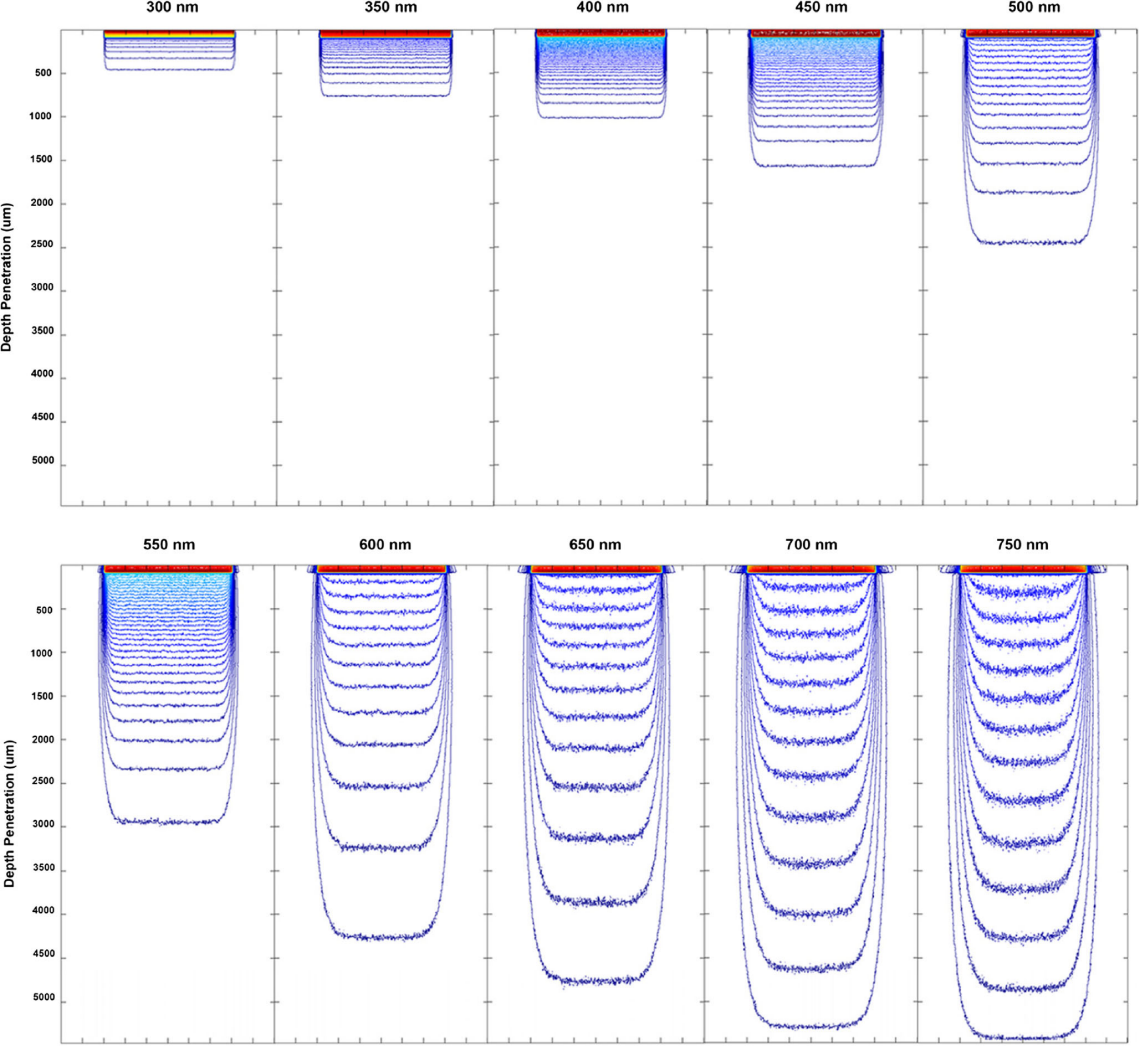
Showing penetration of 300–750 nm photons into tissue matrix from the photon distribution

Monte Carlo simulations were carried out for the whole spectral range offered by the TODDY package; 300–750 nm in increments of 50 nm. Figure 5 clearly illustrates that on increasing the wavelength of light used in skin therapy, there is a corresponding increase in the penetration depth, of the 1% contour lines (which is the line which penetrates the deepest in Fig. 5). Each of the contours represents a 1% fraction of the maximum intensity.

It can be seen that for increasing wavelengths, the 1% contour line penetrates deeper into the tissue as expected. In the penetration depth of 1%, the intensity is reached at 5378 μm (5.4 mm) with a wavelength of 750 nm. This is in agreement with literature which states the penetration of optical light systems reaching to a depth of 4–6 mm [8, 28]. However, it will again be noted that there is a lack of information regarding the definition of penetration depth for light therapy.

**Fig. 6:**
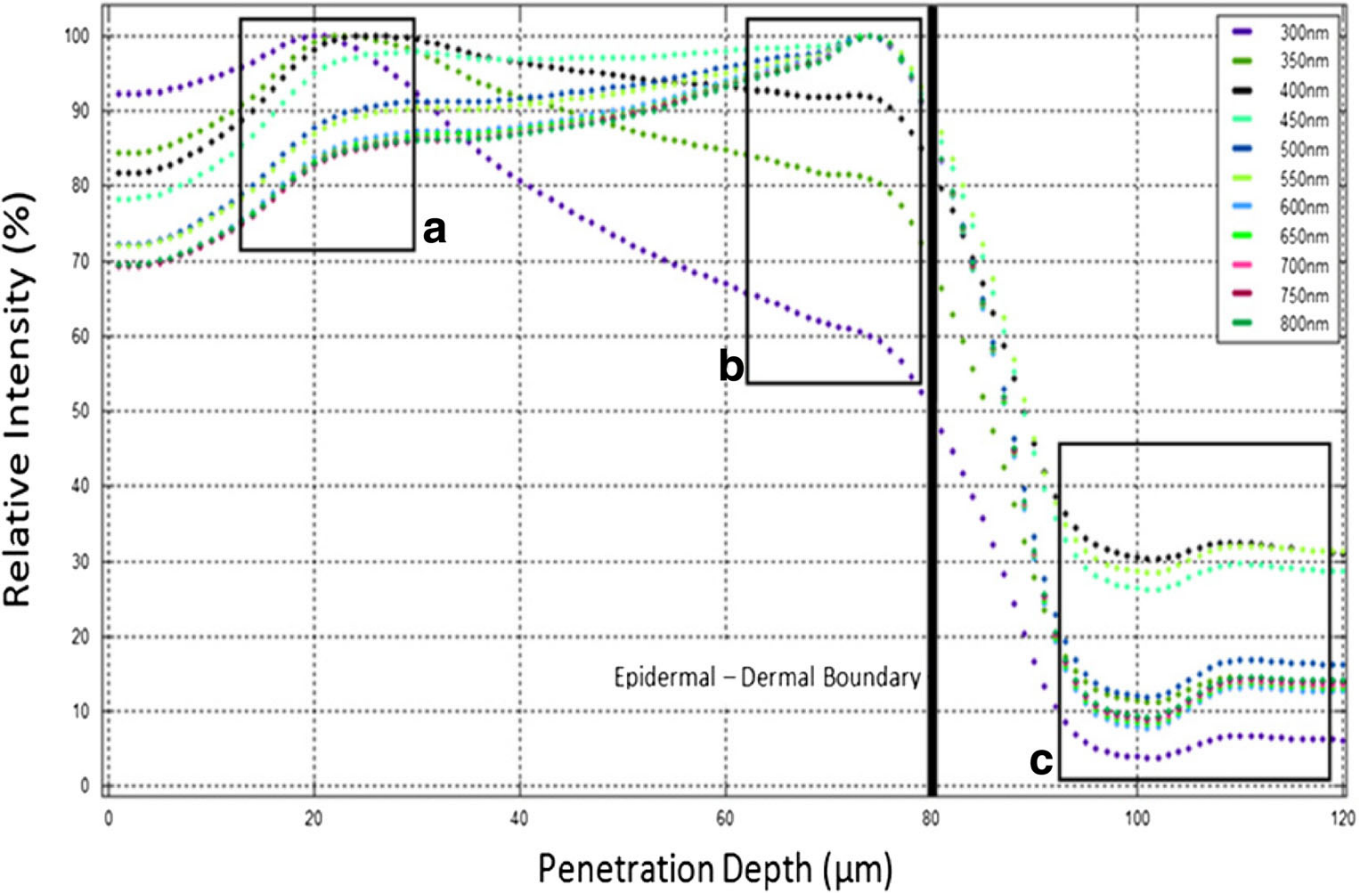
Showing a detailed description of photon deposition for wavelengths ranging 300 to 750 nm

Figure 6 provides a more detailed representation of the wavelength-dependent penetration within the epidermis and the epidermal-dermal boundary. The lower amount of 550 nm absorption is perhaps visualised more clearly here as it passes the epidermal-dermal boundary.

Figure 6 shows that there is greater absorption of shorter wavelength light at the front end of the epidermis (box a) which tails off rapidly towards the boundary layer (b). The increase in intensity of higher wavelengths at b is due to the backscatter from the boundary and the dermal layer into the epidermis which is then absorbed in this region. The backscatter or reflection at the boundary layer is increased by the refractive indices of the epidermis, and the dermis are different and the photons travel from the epidermis to the dermis and back again. Once the light has passed through the boundary layer, its absorption rapidly reduces by greater than 50% for all wavelengths. Again, following the 300 nm curve, this illustrates the highly photoprotective nature of melanin which reduces the blue light absorption by greater than 90% as it passes through into the dermis (c) where the protection from blue light is required most. As can be seen from box c, there is a rapid decrease in the attenuation which then dips and then rises again until a depth of 110 μm is reached. The reason for this behaviour might be explained by the significantly increased mean free path in the dermis relative to the epidermis. It is not known how thick the boundary is, if the boundary consists of a reduced fraction of melanin or if the boundary is simply an abrupt change in attenuation coefficient in the matrix.

## Results—effect of beam width on penetration depth

A key contributor to the penetration depth is the treatment area or spot size employed by the device. Spot size has important clinical implications due to its effect on penetration and light dispersion in tissue. With increasing spot size, there is a reduction in the amount of lateral scattering; this results in greater penetration for larger spot sizes. As a result, lower energy densities can be applied when using larger spot sizes to achieve the same penetration depth for treatment. Variation in spot size is also important depending on the condition being treated, if the treatment region is over a large area then a larger spot size is used and for isolated small lesions in blood vessels for instance, a smaller spot size would be recommended resulting in increased intensity at the target. A beam width of 4–6 mm is usually used to penetrate to the mid-dermal and deeper layers of skin where the hair follicles and blood vessels are situated. It has also been found that although the penetration depth increases, there is a point at which the beam width has no further effect on the penetration depth and this is expected to be in the region of 5–12 mm in beam width [8, 28, 29]. It should also be noted that there is a distinct lack of information regarding the penetration depth for IPL systems with most of the work being carried out on laser treatment devices.

**Fig. 7:**
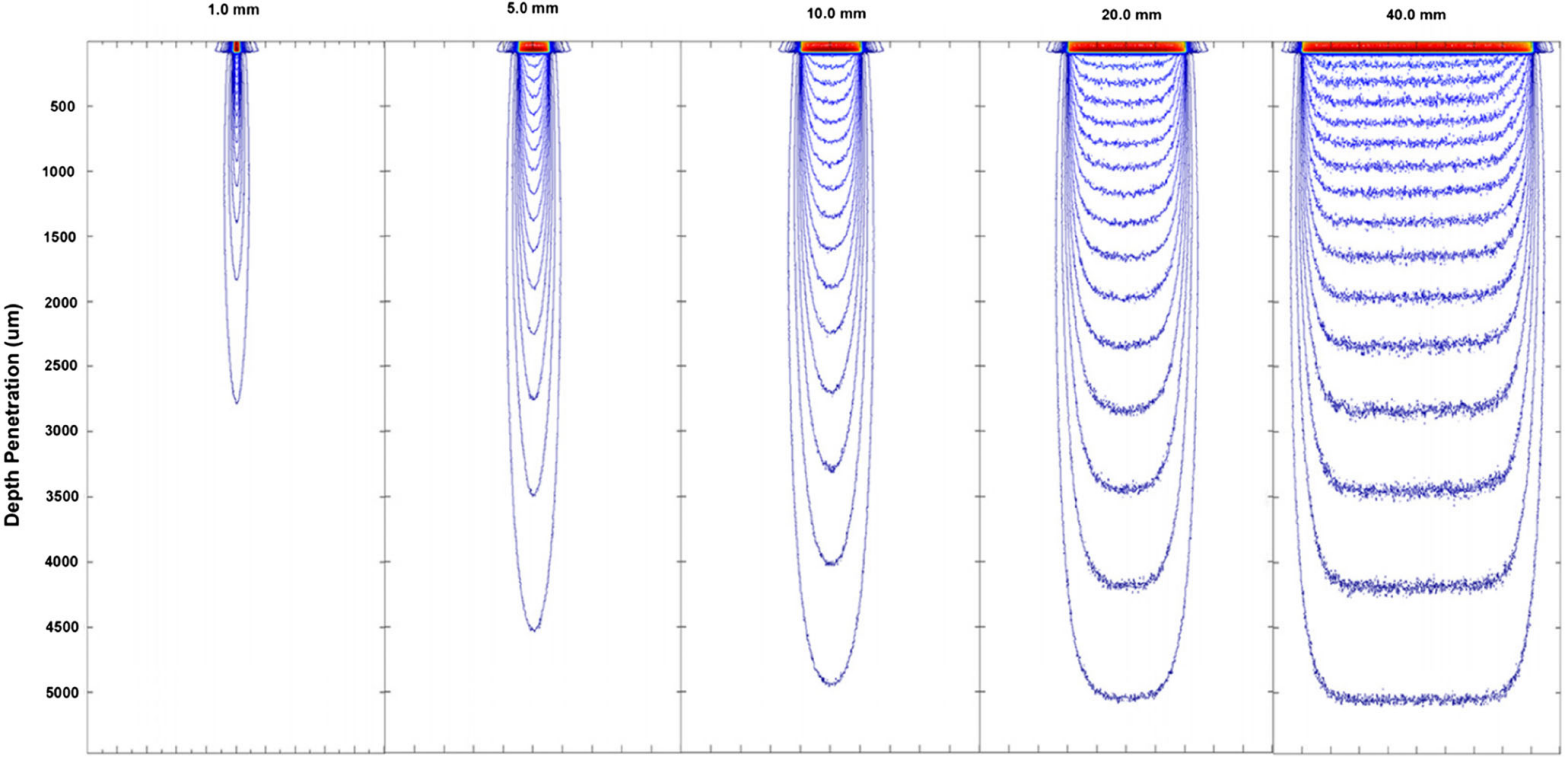
Calculated penetration profiles for uniform 1, 5, 10, 20 and 40 mm width beam, of equal incident fluence obtained by Monte Carlo simulation using typical skin parameters for wavelengths of 525–1100 nm

There is a noticeable increase in the penetration depth when the beam width is increased from 1 up to 5 mm before maintaining a constant penetration depth of approximately 10 mm when the intensity is reduced to 1% regardless of the increase in beam width (Fig. 7).

**Fig. 8:**
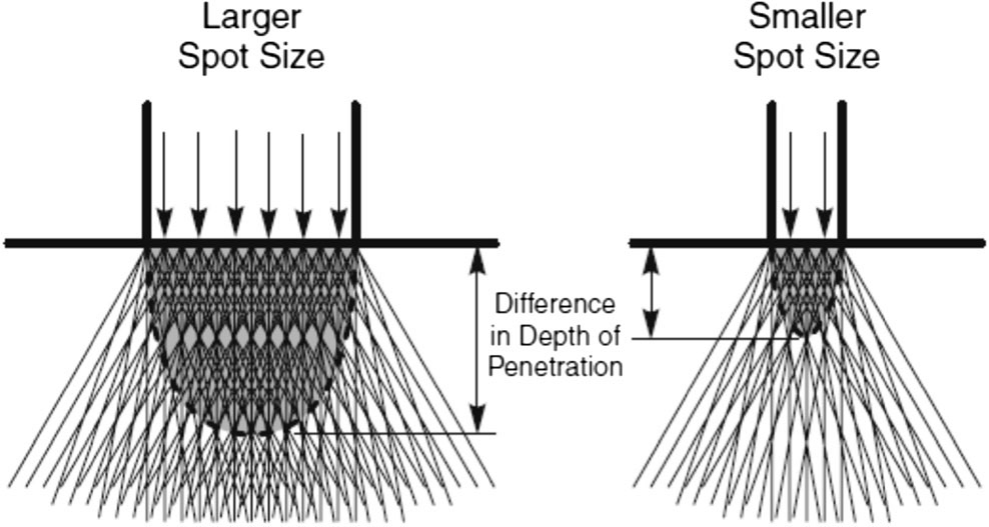
A schematic representation of spot-size-dependent depth of penetration

The trend shows that a critical point is reached for a beam width of 10 mm at which a further increase in the beam width of the treatment device used does not affect the penetration of the light in tissue. A smaller beam width is associated with increased scattering, and as the beam width is increased, the photon propagation becomes increasingly forward projected up to that critical point where the scattering in the medium saturates. This can be graphically shown in Fig. 8.

It was found in the literature that the critical point appears between 5 and 12 mm [29]. There is also a distinct lack of information regarding the therapeutic depth of penetration where papers simply state the penetration depth of light, and it is assumed that by this, it is referring to the maximum depth of penetration, since the criteria used have been found in literature which is not focussed on biological interactions of lasers but simply the theoretical basis of penetration depth.

Beam widths for IPL treatment devices are usually quoted as spot sizes in the range 6 to 4.8 mm [10], where the spot sizes are not always square and thus not always symmetrical, e.g. 20 × 30 mm spot size is employed by the Silk’n system.

Based simply on this 2-D model and the assuming that spot sizes are symmetrical, the fluence levels need not be adjusted for beam widths greater than 10 mm when attempting to reach a specific depth in tissue, and hence, spot sizes for various devices greater than this only have the added benefits of greater area for irradiance and so a reduction in surface heating and also the benefit of a greater beam width to treat a larger volume in the skin.

The Monte Carlo model used is a 2-D model and as such the spot size can only be adjusted in terms of a beam width. Of course, this 2-D model does not give the complete picture of light penetration into the tissue since a typical device may utilise a spatial profile which is not ‘homogeneous’, although it is hoped that the spatial profile remain consistent throughout the medium and does not follow the Gaussian intensity distribution which is employed by laser systems [30-33].

## Discussion

In summary, it has been found from Monte Carlo simulations that an increase in penetration depth can be achieved using longer wavelengths of light and using a spot size which is of at least 10 mm in width. Maximum penetration depths using the IPL system were found to be 5 mm using the 1% criterion and 0.37 mm using the 13.5% criterion. Although from this data and the lack of literature referring to the penetration depth, it is not possible to identify a therapeutic depth of penetration. It can however be suggested that if a maximum penetration depth of 5 mm is achieved using the IPL (for skin type 2) with a reduction in the intensity to the extreme of 1% maximum intensity, then it is safe to assume that the IPL does not have much of an effect at depth greater than 5 mm and so there would be remote risk of damage to tissues beneath the skin and indeed no damage to organs or a foetus which rests beneath the skin, adipose and muscular tissue. However, this model just looks at the penetration depth of light and not at the heat it produces on interacting with the tissue. Thus, it would be useful to produce a spatial heat diffusion model to identify the temperature at the maximum penetration depth and also to identify the temperature rise at therapy associated locations such as the hair follicle and blood vessels.

It should be noted that the intensities reported in this section are not as a percentage of the incident intensity but as a percentage of the maximum deposited photon energy; the radiation which has escaped the sample is not included in this intensity. It would thus be useful for future investigations to identify the percentage of light which has escaped the sample and from this, deduce the absorption intensities as a fraction of the intensity produced by the device and not just as a fraction of that maximum amount which is absorbed in the skin. Therefore, the actual penetration depths would be less than those found.

Other further investigation could include identifying the relationship between wavelength and penetration depth for wavelengths greater than 750 nm, since the Monte Carlo simulation package only looks at wavelengths in the range 300–750 nm. Use of smaller increments would also allow more accurate representation of the wavelength-dependent attenuation profiles of the chromophores to be identified.

It should be acknowledged also that this model does not take into account the compression of skin when the device is placed on it. This would reduce the thickness of skin but at the same time the attenuation coefficients change [1]. However, this may be of irrelevance due to the slight pressure applied when using the device and also the fact that a coupling gel is applied between the device and the skin, though it would be interesting to identify the effect of this compression on penetration depth. Compressing the skin squeezes the blood, a competing chromophore, out of the treatment area and forces the hair follicles to lie down bringing the roots closer to the surface. As a result, the effective penetration of the beam is improved up to 15%.

## Conclusion

This work addresses the risk assessment of laser and IPL devices that at these wavelengths and radiant exposures, photons are unable to reach vital organs as these results show a maximum penetration depth of 5 mm of 1% incident light. Therefore, these devices do not pose a photothermal or photochemical health risk to vital internal organs or foetus’s.

Fluence distribution within tissue and thus the treatment efficacy depends upon the illumination geometry and wavelength. To optimise therapeutic techniques, light-tissue interactions must be thoroughly understood and can be greatly supported by the use of mathematical modelling techniques. The TODDY simulation provides the relationship of photon distribution with wavelength of epidermis-dermis-epidermis backscatter in our model.

This study illustrates that on increasing the wavelength of light used in skin therapy, there is a corresponding increase in the penetration depth. Simulation of various beam widths has presented the increase in the penetration depth when the beam width is increased from 1 up to 5 mm before maintaining a constant penetration depth of approximately 10 mm when the intensity is reduced to 1% intensity regardless of the increase in beam width.
